# Transitions from Hospital‐to‐Home for Dementia with Multimorbidity: A Realist Review Protocol with Preliminary Findings

**DOI:** 10.1002/alz.094670

**Published:** 2025-01-09

**Authors:** Lauren Lawson, Matthew Cooper, Clare Tolley, Hamde Nazar

**Affiliations:** ^1^ Newcastle University, Newcastle Upon Tyne United Kingdom

## Abstract

**Background:**

Older adults with dementia live with an average of four additional long‐term conditions (multimorbidity), and experience frequent transitions between fragmented care settings. This can compromise their safety due to an increased risk of miscommunication, errors, and delays in receiving appropriate treatment. Unsafe transitions are also associated with increases in mortality, morbidity, and preventable readmissions. The experiences of adults managing dementia alongside multimorbidity has not often been reflected in the literature. As such, it is currently unclear how dementia, alongside existing multimorbidity can influence transitions from hospital‐to‐home, and what currently works in this process. This review aims to synthesise existing literature to understand and explain how, to what extent, and under which circumstances transitions from hospital‐to‐home work for older adults with dementia and multimorbidity.

**Method:**

This review was registered on PROSPERO and follows Realist and Meta‐narrative Evidence Syntheses: Evolving Standards. Following on from the development of an initial programme theory, six electronic databases (CINAHL/Embase/MEDLINE/PsycINFO/PubMed/Scopus/Web of Science) and two grey literature databases (HMIC/ProQuest Dissertations and Theses) were systematically searched using key terms. Eligible documents included adults aged 65 and over with dementia and at least one other long‐term condition, informal caregivers or healthcare professionals involved in their care, and investigated transitions from hospital‐to‐home. Research investigating end‐of‐life care or aged‐care facilities was excluded. Three additional, independent reviewers screened 10% of documents at each level for consistency. Realist quality appraisal will assess relevance and rigour of included documents. Deductive and inductive coding will be performed to identify prominent interactions between contexts, outcomes, and causal mechanisms.

**Results:**

This review is iterative and ongoing. The first search identified 4,396 (database) and 43 (grey) articles. From 349 documents screened at full‐text, 73 are being reviewed for inclusion. Preliminary evidence suggests that the extent to which a transition from hospital‐to‐home works is influenced by trust, effective communication, awareness of dementia, health literacy, inconsistent use of digital health technologies, and prioritisation of non‐acute patients with dementia in hospital.

**Conclusions:**

The final, revised programme theory and context‐mechanism‐outcome configurations produced upon completion of data synthesis will be used to develop suitable recommendations to inform improvements in transitional care pathways.

